# Genome Sequences of the First WHO Repository of Platelet Transfusion-Relevant Bacterial Reference Strains

**DOI:** 10.1128/genomeA.00001-17

**Published:** 2017-07-20

**Authors:** Alexander Mellmann, Eva Spindler-Raffel, Stefan Bletz, Marcel Prax, Isabelle Bekeredjian-Ding

**Affiliations:** a Institute of Hygiene, University Hospital Muenster, Muenster, Germany; b Paul-Ehrlich-Institute, Federal Institute for Vaccines and Biomedicines, Microbial Safety Section, Langen, Germany

## Abstract

To develop novel techniques for improving blood safety, dedicated bacterial strains, which are able to persist and to proliferate in blood platelet concentrates, are needed. Here, we present draft genome sequences of the four bacterial strains approved for the first WHO repository of platelet transfusion-relevant bacterial reference strains.

## GENOME ANNOUNCEMENT

The bacterial contamination of blood platelet concentrates (PCs) remains a problem in blood transfusion medicine. In spite of the improvements made over the last decade to reduce the frequency of transfusion-transmitted bacterial infections—such as the introduction of closed systems, the improvement of techniques for skin disinfection, and the diversion of first blood volume at the time of donation—bacterial contamination of PCs is still the major posttransfusion infectious risk (1–8). Under the usual storage conditions of PCs at 20 to 24°C with agitation, bacteria contaminating a PC can grow from very low initial titers up to 10^10^ CFU per bag (9). Different measures to increase blood safety, such as the improvement of bacterial detection methods (i.e., automated culture systems or pathogen reduction technologies), offer a proactive approach to preventing bacterial contamination. The availability of bacterial reference strains will allow regulatory agencies as well as the manufacturers of technologies for blood safety to decide on those approaches in an objective and standardized manner. Therefore, the International Society of Blood Transfusion (ISBT) TTID Working Party on Bacteria together with the Paul-Ehrlich-Institute (Germany) initiated an international study on platelet transfusion-relevant bacterial reference strains (PTRBRs). Due to the fact that growth ability may vary among bacterial species, even at the strain level, it is important to validate the candidate strains in an international collaborative study. The outcome of this study was the establishment of the first WHO repository of PTRBRs (4, 10). The bacterial panel includes the strains *Staphylococcus epidermidis* PEI-B-P-06, *Klebsiella pneumoniae* PEI-B-P-08, *Streptococcus pyogenes* PEI-B-P-20, and *Escherichia coli* PEI-B-P-19. These four strains are cultivated and distributed by the Paul-Ehrlich-Institute (PEI code no. 8483/13).

The whole genomes of these four strains were sequenced using Nextera XT chemistry (Illumina, Inc., San Diego, CA, USA) either for a 100-bp paired-end sequencing run on an Illumina HiScanSQ sequencer or for a 250-bp paired-end sequencing run on an Illumina MiSeq sequencer. Subsequent quality trimming and *de novo* assembly were performed as described recently (11).

As a basic set of molecular typing (none of the strains exhibited a remarkable phenotype), we extracted, if possible, the species-specific multilocus sequence types (STs) and ribosomal STs (rSTs) (7) from the isolates. These were ST626 and rST46853 for *Staphylococcus epidermidis* PEI-B-P-06, ST48 and rST19218 for* Klebsiella pneumoniae* PEI-B-P-08, ST28 (genes for rST determination were not completely present in the genome sequence) for* Streptococcus pyogenes* PEI-B-P-20, and ST3854 and rST30111 for* Escherichia coli* PEI-B-P-19.

### Accession number(s).

This whole-genome shotgun project has been deposited in ENA under sample accession numbers FUVD00000000, FUVE00000000, FUVF00000000, and FUVG00000000.
